# Genome Variations Associated with Viral Susceptibility and Calcification in *Emiliania huxleyi*

**DOI:** 10.1371/journal.pone.0080684

**Published:** 2013-11-19

**Authors:** Jessica U. Kegel, Uwe John, Klaus Valentin, Stephan Frickenhaus

**Affiliations:** 1 Alfred Wegener Institute for Polar- and Marine Research Bremerhaven, Bremerhaven, Germany; 2 Hochschule Bremerhaven, Biotechnology, Bremerhaven, Germany; American University in Cairo, Egypt

## Abstract

*Emiliania huxleyi*, a key player in the global carbon cycle is one of the best studied coccolithophores with respect to biogeochemical cycles, climatology, and host-virus interactions. Strains of *E. huxleyi* show phenotypic plasticity regarding growth behaviour, light-response, calcification, acidification, and virus susceptibility. This phenomenon is likely a consequence of genomic differences, or transcriptomic responses, to environmental conditions or threats such as viral infections. We used an *E. huxleyi* genome microarray based on the sequenced strain CCMP1516 (reference strain) to perform comparative genomic hybridizations (CGH) of 16 *E. huxleyi* strains of different geographic origin. We investigated the genomic diversity and plasticity and focused on the identification of genes related to virus susceptibility and coccolith production (calcification). Among the tested 31940 gene models a core genome of 14628 genes was identified by hybridization among 16 *E. huxleyi* strains. 224 probes were characterized as specific for the reference strain CCMP1516. Compared to the sequenced *E. huxleyi* strain CCMP1516 variation in gene content of up to 30 percent among strains was observed. Comparison of core and non-core transcripts sets in terms of annotated functions reveals a broad, almost equal functional coverage over all KOG-categories of both transcript sets within the whole annotated genome. Within the variable (non-core) genome we identified genes associated with virus susceptibility and calcification. Genes associated with virus susceptibility include a Bax inhibitor-1 protein, three LRR receptor-like protein kinases, and mitogen-activated protein kinase. Our list of transcripts associated with coccolith production will stimulate further research, e.g. by genetic manipulation. In particular, the V-type proton ATPase 16 kDa proteolipid subunit is proposed to be a plausible target gene for further calcification studies.

## Introduction

The prolific coccolithophore *Emiliania huxleyi* is distributed from sub-polar to tropical latitudes [1], [2] and often forms immense coastal and open ocean blooms that can cover more than 50.000 km^2^ [3], [4], [5]. These blooms can be detected via satellite imagery due to the reflection of the calcite platelets, the coccoliths [6], [7]. This makes *E. huxleyi* an important component within the global biogeochemical cycles of carbon and sulphur and one of the most important species on earth with respect to sediment formation and climate [8], [9]. Therefore, it is a key species for current studies on global biogeochemical cycles [10]. It is also of interest to scientists from fields as diverse as geology, biogeography, paleoclimatology, ecophysiology, material science, and medicine [11]. Whereas bloom formation is mainly stimulated by abiotic factors, bloom control and termination is often influenced by viral infection [12], [13]. A range of viruses specific to *E. huxleyi* (EhVs) have been isolated [14], [15] and were further analyzed for their phylogeny [16], [17], genome structure of *Emiliania huxleyi* virus 86 (EhV-86) [18], [19], [20], and ecological succession in mesocosm experiments [21], [22] and in nature [23]. Hence, it is one of the best studied eukaryotic phytoplankton host-virus systems to date [24], [25]. Previous studies have reported different genome sizes among morphotypes of *E. huxleyi* from different geographical regions via restriction fragment length polymorphism (RFLP) analysis [26], DNA microsatellites, [27], cox1b-atp4 genes [28], and *tufa* [29]. Results indicate the presence of different ecotypes of *E. huxleyi* with differences in genome organization as a response to environmental conditions or potential threats, such as viral infections. Furthermore, results show the lack of variation in coding (18S/16S) and non-coding (ITS/Rubisco) regions of *E. huxleyi* and its closest relative *Gephyrocapsa oceanica,* from which *E. huxleyi* separated about 270,000 years ago [30].

A genotype can develop multiple phenotypes depending on environmental conditions [31]. This so-called phenotypic plasticity is widespread in nature and can alter numerous interactions between organisms and their biotic and abiotic environments [32]. Furthermore, examples for a connection between genetic variation and virus susceptibility have been demonstrated [16], [33]. It was found that virus resistant strains of *E. huxleyi* display a higher dimethylsulfoniopropionate lyase (DMSP-lyase) activity than strains that are susceptible to virus infection. One reason for the different enzyme activities could be variations in expression or copy numbers of genes coding for DMSP lyase. Furthermore, Bidle and Kwityn [33] showed a connection between caspase activity and virus susceptibility. Resistant *E. huxleyi* strains were characterized by low caspase activity while sensitive strains had elevated caspase activity.A microbial genome can be partitioned into core and variable parts, both parts together making up the pan-genome [34]. The core genome is composed of genes which are present in all strains of a species and typically comprise genes necessary for basic metabolism [35], [36]. The variable genome consists of a set of genes which are detected in only some strains, and enable these strains to thrive in particular niches [36], [37], [38].

Over the last decade, comparative genomic hybridization (CGH) has been extensively utilized to elucidate genetic diversity between the genomes of closely related taxa, such as species and strains. CGH was mainly applied in bacterial systems, including *Helicobacter pylori*, *Campylobacter jejuni*, *Francisella tularensis*, and *Escherichia coli* among others [39], [40], [41], [42] but also in a eukaryote (yeast [39]). Results indicated that polymorphism in gene content is not uncommon, suggesting genetic adaptations to different ecological niches [39], [40], [41].

Here, we provide insights into the genetic diversity and genome evolution of a key player in marine phytoplankton, *E. huxleyi*, by CGH of 16 strains. We used a whole genome microarray comprising unique probes for predicted gene models of the sequenced strain *E. huxleyi* CCMP1516 (reference strain). This analysis gives information about a shared pool of genes (core genome) as well as about genes specific for the sequenced reference strain CCMP1516 (genome project conducted by JGI and lead by Betsy Read, http://genome.jgi-psf.org/Emihu1/Emihu1.home.html, [42]). Within the variable genome, we were able to identify candidate genes possibly involved in virus susceptibility and calcification.

## Results and Discussion

### Core Genome and Genetic Diversity of E. huxleyi

**Figure 1:**
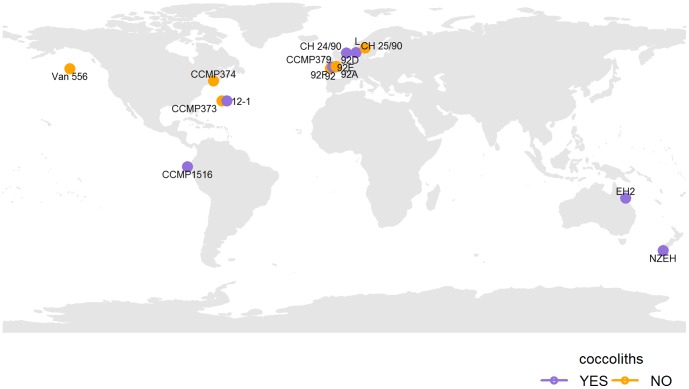
Isolation sites of the 16 E. huxleyi strains. World map depicting the isolation sites of the 16 E. huxleyi strains and wether they possess coccoliths (blue) or not (orange).

Genomic DNAs of 16 *E. huxleyi* strains from different geographic regions (Figure 1) were compared in order to identify genomic differences in terms of plasticity and possible relation to virus-susceptibility and calcification. Comparative genomic hybridization (CGH) was used to characterize 15 strains with respect to gene content similarities by using the sequenced virus susceptible and calcifying *E. huxleyi* strain CCMP1516 as the reference.

Hybridization intensities were analysed statistically to determine either the presence, expressed as relative copy number of each gene transcript, or its absence or modification, indicated by the absence of cross-hybridisation. *Gephyrocapsa oceanica* and *Isochrysis galbana,* phylogenetically closely related taxa, were used as outgroup.

**Figure 2:**
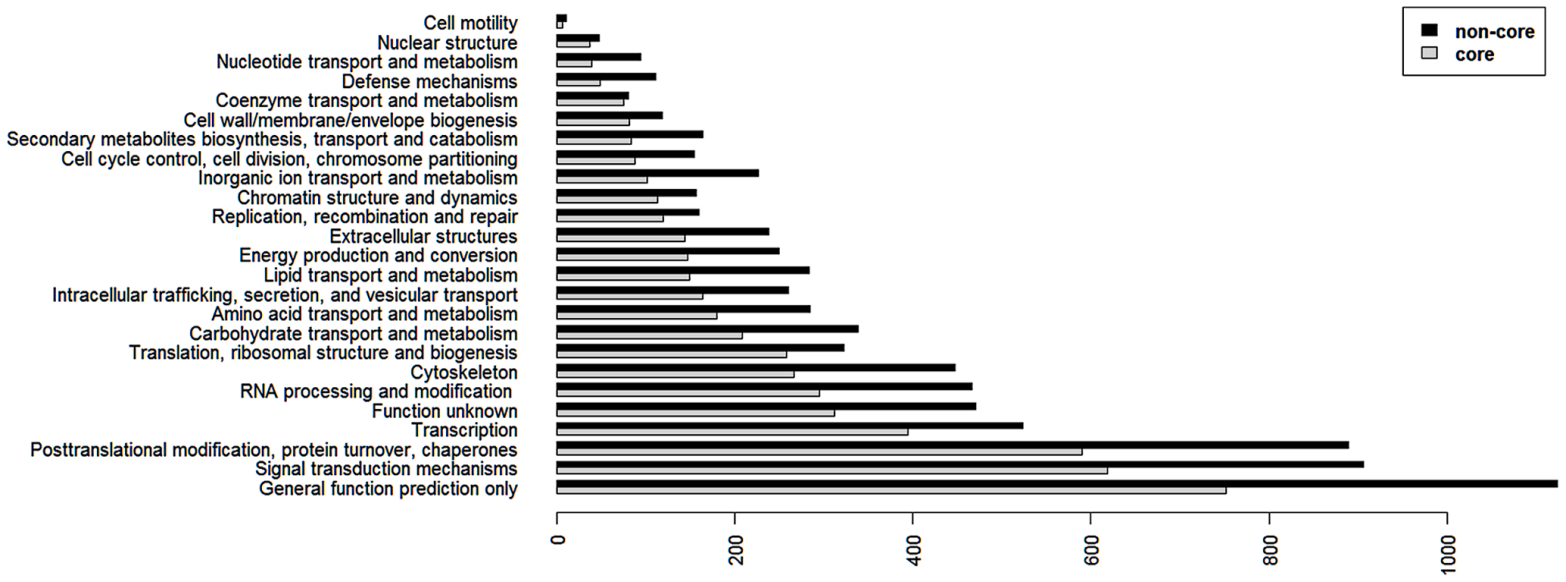
Distribution of KOG annotations and comparison of the core and non-core (variable) genome of (16 strains of) E. huxleyi. KOG annotations of the E. huxleyi genome strain CCMP1516 (JGI) were used to identify functional classes of the core and non-core genome.

To check for possible unspecific probes, ANOVA was used (p<0.01). The obtained 31940 probes, representing gene models as identified in the genome by JGI, were classified as present or modified/absent genes by using the software GACK in its standard configuration [43]. By looking at the intersection of all genes showing 95% estimated probability of presence, we identified 14628 core genes in all 16 investigated strains (Figure 2, Table S1). As only slight modifications within a gene may result in absence of a hybridisation signal we regard this as a minimal number of core genes. Likely more genes belong to the core genome but this could only be clarified by sequencing of these strains.

We compared functional coverages of core and non-core genomes, respectively, using KOG statistics (Figure 2). Distribution of functions for both gene sets is quite similar, i.e. no KOG category is over-represented in either fraction. Surprisingly we found that genes of the variable genome (non-core) represented more KOG functions in all categories than core genes. This coincides with a higher number of annotated genes among the non-core genes versus the core genes. We expected this to be the opposite as the “core-genome” should contains house keeping genes necessary in all strains, and consequently genes better represented in the database and more easy to identify. For the strain specific genes we expected a high content of genes necessary only under particular conditions and as such less well known. The difference is not accounted for by different numbers of KOG hits per gene for core versus non-core genes. One possible exception is the category of coenzyme transport and metabolism where both group produced an almost equal number of KOG hits. The highest relative difference between the variable genome and the core genome is found in the category of defence mechanisms, implying a large variability of such genes among strains. Defence in general is “costly” and specific defence less often required than house keeping genes. Therefore, such genes are probably deleted more quickly from the core than others. Core genes among all analysed isolates are probably necessary for general survival in diverse environments where the species is found. The remaining specific set may reflect for the ‘niche’ adaptations of the different isolates, and for specific defence mechanisms.

Besides overlapping genes (i.e. those found in more than one strain) we identified 224 strain-specific probes representing 224 gene models for the reference genome from strain CCMP1516 (Table S2). This number could be an over-estimation as an oligo might not bind to an existing homologous gene only because 1–2 bases mismatch. Functions could be assigned to 74 genes as identified by the probes. According to KEGG classification (http://www.genome.jp/kegg/), these reflect proteins involved in metabolism, folding, sorting and degradation, replication and repair, cellular processes, transport and environmental information processing among the reference strain specific gene set. The remaining 150 genes had no significant similarity to sequences in the public sequence databases or were of unknown function.

**Figure 3:**
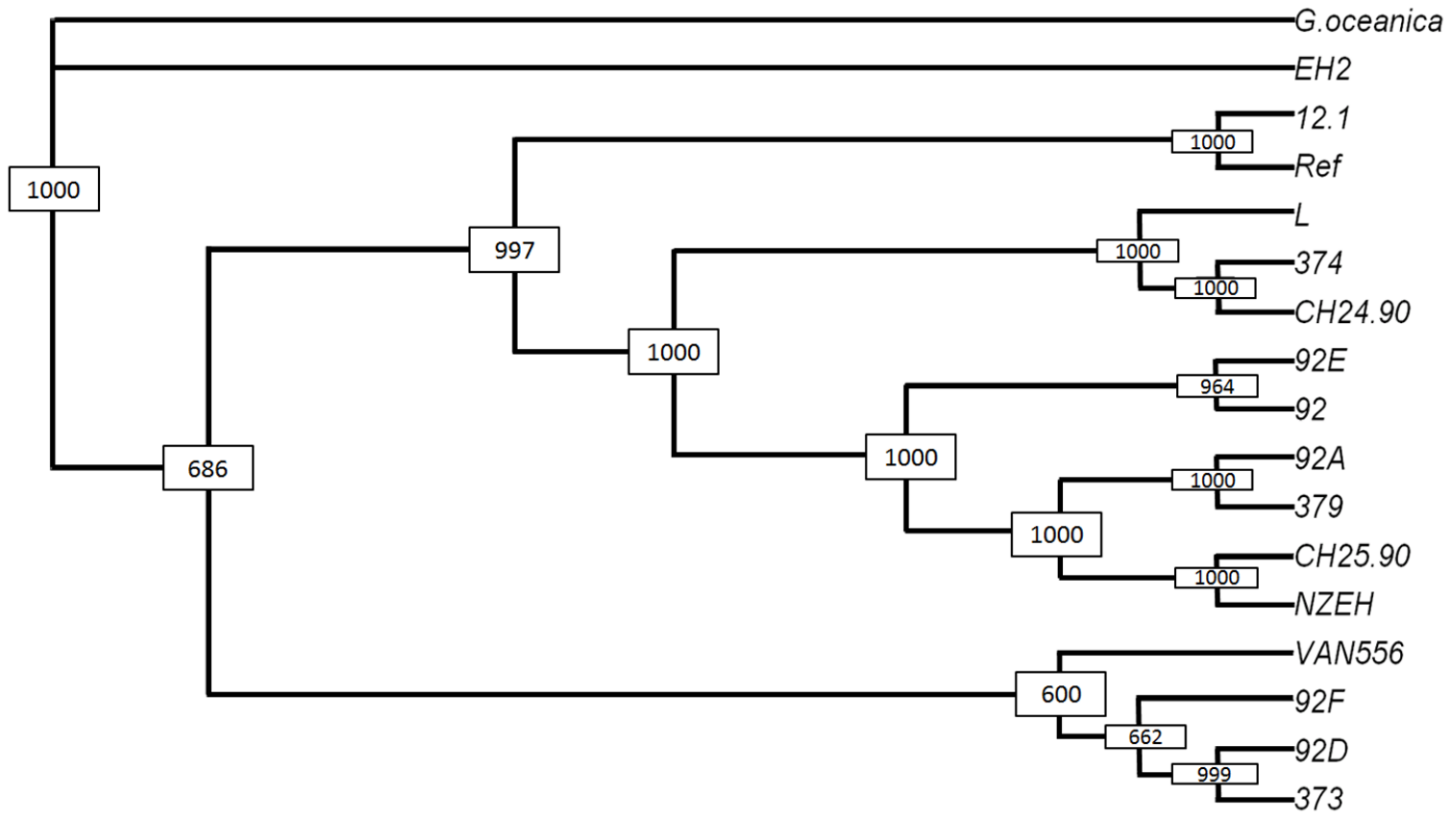
Bootstrapped neighbour-joining consensus dendogram of the 16 E. huxleyi strains and G. oceanica. Dendrogram with bootstrap values obtained from 1000 replicates of 16 E. huxleyi strains and G. oceanica as outgroup. Distances were computed from a matrix of ones (gene present with probability >95%) and zeros (rest).

A bootstrapped neighbour-joining consensus dendrogram of the 16 *E. huxleyi* strains and the out-group *G. oceanica* based on the CGH data is depicted in Figure 3. As expected, the out-group is separated from all *E. huxleyi* strains with 100% bootstrap (BT) support. Interestingly, the *E. huxleyi* strain EH2 also is separated from other *E. huxleyi* strains by BT support. The remaining 15 *E. huxleyi* strains grouped into two main clusters. The strains Van556, 92D, 92F and CCMP373 showed the highest degree of divergence to all other *E. huxleyi* strains and clustered into one of the main groups. The second main cluster can be subdivided into sub-groups on different scales. Strain 12-1 was most similar to the reference strain (89.1% identity in gene contents) and clustered into one of the five sub-groups.

The analysis of hybridization patterns showed that the gene dissimilarity between strains ranged from 10,9% to 30,1% (3015 genes differed, compared to 31468 positive reference hybridization signals). The variation exhibited in these genes is probably associated with (partial) gene deletion, nucleotide sequence divergence or gene duplication. When comparisons were made between the outgroup *G. oceanica* and the reference, the genetic distance increased only slightly up to 30,4% (9232 genes, summarized in Table S1).

So far, genome comparative studies of microorganisms using CGH were restricted to bacteria such as *E. coli* [44], *Bacillus subtilis* [45], and *Streptococcus* [36] and to yeast [39]. Fewer studies exist of genome comparisons in marine environments (*Vibrio cholerae* [46], *Ectocarpus siliculosus* [47]). Genome comparisons of strains for a given species in these studies showed differential hybridization between 0.17 and 16.7 per cent of the gene transcripts.

In contrast, our results revealed between 10.9 and 30.1% gene variation within the species and up to 30.4% compared to the genus *G. oceanica*, their last common ancestor. As *E. huxleyi* has evolved from *G. oceanica* only 268.000 years ago [30] and became dominant around 70.000 years ago, this gene diversity indicates that *E. huxleyi* is undergoing a more rapid evolutionary radiation than other species and is better described as a species-morpho-complex than as a single species. This could also explain its enormous phenotypic plasticity described in the literature [48], [49], [50].

The highest variability amongst the strains was observed for strain 1516 against strain EH2 (30.1%), almost as much as between the genera *Gephyrocapsa* and *Emiliania* (30.4%). Strain EH2 is virus susceptible and can produce coccoliths as the reference strain (CCMP1516). The reasons for the high genomic deviation from the reference could be many fold, including its different geographic origin, ecological niche, and predation (grazing, virus infection etc.) The reference strain was isolated near the coast of Ecuador whereas EH2 was obtained from the Great Barrier Reef (Table 1, Figure 1). Clearly differences in the ecological and life cycle strategies of *E. huxleyi* strains (e.g. bloom dynamics) could also cause these gene differences [51]. Results of the recent study of Cook et al (2011) using *tuf*A as a molecular marker support our study showing two distinct clades (Bayesian analysis): one of the southern hemisphere and one showing *G. oceanica* and *E. huxleyi* together suggesting inter-breeding between the two genera (Linda Medlin, personal comment) which could be hypothesized for our case.

**Table 1:** Isolation sites and date of the 16 E. huxleyi strains and whether they possess coccoliths (Y  =  yes) or not (N).

Emiliania huxleyi strain	Coccoliths	Collection site	Isolation date
92 (English Channel)	N	49°19N 07°26W	1950
92A (English Channel)	N	50°10N 4°15W 1mile west of Eddystone	1957
92D (English Channel)	Y	50°02N 4°22W	1975
92E (English Channel)	Y	49°52N 06°12W 2m depth	1992
92F (English Channel)	Y	49°52N 06°12W 2m depth	1992
CCMP379 ( = 92A, according to CCMP)	N	50°10N 4°154W 1mile west of Eddystone	1992
CCMP374 (Gulf of Maine)	N	42°30N 69°W Gulf of Maine (5 meters)	1989
CCMP373 (Sargasso Sea)	N	32°10N 64°30W	1960
12-1 ( = CCMP371) (Sargasso Sea)	Y	32°00N 62°00W (50 meter depth)	1987
CCMP1516 (South Pacific)	Y	2°40S 82°43W (surface)	1991
Van 556 (North Pacific)	N	49°05N 144°40W	1984
CH 24/90 (North Atlantic)	Y	57°20N 01°09E	1990
CH 25/90 (North Atlantic)	Y	57°26N 6°13E	1990
L (Oslo Fjord)	N	60°N 11°E	1959/68/80
NZEH (South Pacific)	Y	Big Glory Bay, NZ	1992
EH2 (South Pacific)	Y	Great Barrier Reef	1990

Other reasons for the genetic distance between these two strains include different genome size. Read *et al.* [42] report on genome sizes ranging from 99 to 133 Mb (haploid) for different strains of *E. huxleyi* (Supplementary material to [42]: Table 6). They also estimate numbers of missing genes, compared to the reference. This documents that genome size is very likely to show a signature in genetic distance in terms of gene function repertoire.

As highlighted above, *E. huxleyi* is an important calcifier in the ocean, and at the same time non-calcifying strains exist. Its immense blooms are often terminated by viruses. Therefore, within the variable genome, we searched for specific genes which may be involved in virus susceptibility and coccolith production. The 15 compared *E. huxleyi* strains were divided into the following groups: virus susceptible vs. virus resistant (Table 2) and possession of coccoliths vs. no possession of coccoliths (Table 1), genes specific for either group were identified.

**Table 2:** Virus susceptibility and resistance of E. huxleyi strains derived from Allen et al. [18].

	*Emiliania huxleyi* virus (EhV) strain								
*Emiliania huxleyi* host strain	86	84	88	163	201	205	202	208	207
92 (English Channel)	–	–	–	–	–	–	–	–	–
92A (English Channel)	–	–	–	–	–	–	–	–	–
92D (English Channel)	–	–	–	–	–	–	–	–	–
92E (English Channel)	–	–	–	–	–	–	–	–	–
92F (English Channel)	+	+	+	+	+	+	+	+	+
CCMP379 (Unknown)	–	–	–	–	–	–	–	–	–
CCMP374 (Gulf of Maine)	+	+	+	+	+	+	+	+	+
CCMP373 (Sargasso Sea)	–	–	–	–	–	–	–	–	–
12-1 (Sargasso Sea)	–	–	+	+	+	+	+	+	+
CCMP1516 (South Pacific)	+	+	+	–	+	+	+	+	+
Van 556 (North Pacific)	–	–	–	–	–	–	–	–	–
CH 24/90 (North Atlantic)	–	–	–	–	–	–	–	+	–
CH 25/90 (North Atlantic)	–	–	–	–	–	–	–	–	–
L (Oslo Fjord)	+	+	+	+	+	–	+	+	+
NZEH (South Pacific)	–	–	–	–	–	–	–	–	–
EH2 (South Pacific)	+	+	+	–	+	+	+	+	+

+, culture lysis; –, no evidence of lysis after 14 days of viral infection cultures were not lysed and considered to be non-susceptible to the virus strain [18].

### Genes involved in virus susceptibility

The *E. huxleyi* EhV system has emerged as an excellent model to understand the biochemical “arms race” in the oceans (summarized by [25]). It involves infection-induced ROS production [52] and subsequent caspase-induced programmed cell death (PCD) controlled by the virus [24]. *E. huxleyi* possibly counter-acts by dimethylsulfoniopropionate (DMS) and acrylic acid release which can scavenge oxygen radicals [53]. Glycosphingolipids (GSL) seem to play an important role in the process in that the virus controls synthesis of viral myristoyl-based GSL, different from the host palmitoyl-based SPL, which in turn control host metabolism and EhV production; viral GSL can even induce PCD [23]. Viral GSL are likely also present in the viral envelope which could be used for a fusion mechanism to enter the host cell, similar to the situation in many animal viruses.

The analysis of gene contents correlated to virus susceptibility yielded 94 genes (Table S3) linked to this trait. More than half of these (54) showed no similarity to any sequences in public sequence databases or were of unknown function. According to KEGG (http://www.genome.jp/kegg/) classification we found proteins involved in metabolism (12), transcription and translation (7), transport (5), cellular processes and signalling (4), carbohydrate and lipid metabolism (4), genetic information processing (2), signal transduction (4), and folding, sorting, and degradation (2). Within these groups, a Bax inhibitor-1 like protein (BI-1) and ten different protein kinases, including three leucine-rich repeat (LRR) receptor-like protein kinases and one mitogen-activated protein kinase (MAPK) were identified and might be of particular interest.

The identification of BI-1 (Figure S1) is rather plausible, as this protein might serve as a cell death regulator protein that inhibits Bax induced cell death as has been shown by Hückelhoven [54], [55]. BI-1 is a conserved protein found in all organisms including plants and fungi. Even viruses code for proteins with a domain architecture similar to BI-1 indicating that BI-1 has been corrupted during evolution by pathogens to reprogram a living host cell [54]. Eukaryotic cells permanently have to cope with environmental cues and to integrate developmental signals. Cell survival or death is the possible outcome [56]. Likely most *E. huxleyi* blooms are terminated by viral infection via virus-induced apoptosis, as a form of (PCD) [24]. Indeed, as Bidle et al (2007) showed the virus rather makes use of and induces PCD in *E. huxleyi* for its benefit and proliferation rather than the host inducing PCD to terminate virus proliferation. Recently, it has been shown that BI-1 is required for full susceptibility of barley to powdery mildew, suppressing the defense response of the host [57]. Accordingly BI-1 could therefore well be a susceptibility factor of *E. huxleyi* strains and involved in virus-induced cells apoptosis.

LRR motifs are found in many plant and animal proteins and are usually involved in protein-protein interactions and ligand binding [58]. Receptor-ligand interactions are very sensitive to point mutations of the DNA-sequence, which can lead to viral resistance/or can allow pathogens to avoid recognition [59]. LRRs are also found in the human Interleukin-1 and Toll-like receptors, which participate in the regulation of immune responses [60], [61]. The identification of MAPK is consistent with recent observations of Marchant et *al*. [60], which have shown that MAPK is a determinant of virus infection even knowing that the MAPK pathway is involved in many substantial regulative processes (see [62]). It has furthermore been shown, that the vaccinia virus replication requires the MAPK pathway [63].

Animal dsDNA enveloped viruses like herpes simplex virus (HSV) and vaccinia virus enter their host either via clathrin-mediated endocytosis or by fusion with the plasma membrane [64]. Both processes involve the fusion of the virion envelope with a cell membrane, either the plasma membrane or a vesicle membrane. In general, the first step of virus infection involves attachment of virus particles to host-specific cell surface receptors [65], [66] prior to entering the host cell. Once inside the host cell, viruses utilize the host machinery in order to enhance the efficiency of its replication process. This is of particular importance for EhV86 which encodes hundreds of genes as compared to only a handful in ssRNA animal viruses. Consequently, the expression of a receptor on the outer surface of the host is a major determinant of the route of entry of the virus into the host and of the patterns of virus spread and pathogenesis in the host [65]. Viruses have evolved to exploit these receptors to gain entry into cells. As each virus is looking for only one specific receptor that fits its attachment protein, the host receptor will, in part, determine the susceptibility of different hosts to the same virus. Previous studies have demonstrated that the lack of receptor expression restricts virus entry [67], [68], [69] and that protein kinases influence virus entry and infectivity [70], [71], suggesting that the LRR receptor-like protein kinase as well as MAPK and serine/threonine protein kinase could be involved in virus susceptibility, infection or induced defence mechanisms. We have identified three leucine-rich repeat (LRR) receptor-like protein kinases and MAPK among the genes present only in virus-susceptible *E. huxleyi* strains. These genes suggest the route of entry of the virus into the host for the virus-susceptible group and may be similar to above described animal virus infection pathways. It also shows that the lack of them due to possible point mutations tends to lead to resistance to such viruses, possibly by avoidance of recognition.

Our analysis is limited by the availability of only one sequenced *E. huxleyi* genome which is from a virus susceptible strain. Therefore we can not identify possible resistance genes in the resistant strains due to the limitation of our microarray design. We therefore regard it as very likely that virus susceptibility of *E. huxleyi* may be dependent on the expression of other genes or factors for viral entry. However, differences in copy numbers or point-mutations of coding sequences of the identified receptor-like protein kinases could be an indication for differences in virus susceptibility, making them suitable targets for further studies.

### Genes involved in calcification

A total of only 11 genes were identified as possibly associated with calcification (Table S4). Three of them showed no similarity to sequences in the public sequence databases or were of unknown function. We identified a kelch-like protein, one activator of 90 kDa heat shock protein ATPase homolog, and one uncharacterized oxidoreductase. Moreover, we identified one arylsulfatase which binds one Ca^2+^ ion per subunit suggesting a potential role in calcification. A long-chain-fatty-acid-CoA ligase was identified, which is involved in the lipid metabolism. The identification of two V-type proton ATPase 16 kDa proteolipid subunits is of high relevance as these are involved in transport and were also found in the calcifying coccolithophore *Pleurochrysis carterae* [72]. Studies of calcification in the coccolithophore *P. carterae* have previously localized a vacuolar H^+^-ATPase (V-ATPase) in the coccolith vesicle mediating Ca^2^^+^/H^+^ exchange [72], [73], [74]. Moreover, recent transcriptome analyses have shown an over-expression of numerous V-ATPase subunits in diploid calcifying cells [75], [76] indicating putative roles in the calcification process as reviewed by Mackinder *et al*. [77].

Coccolith formation and structure has been extensively studied in the species *Emiliania huxleyi* [77], [78], *Pleurochrysis carterae* [79] and *Coccolithus pelagicus* [80]. Formation of coccoliths takes place in a Golgi-derived intracellular vesicle [81], [82] before transport to the cell cortex and secretion to the cell surface in a single exocytotic extrusion event [80]. In *Pleurochrysis* coccolith precursors are mediated by acidic polysaccharides [81], [83]. However, despite the existence of several hypotheses (for an overview see Young [84] and Mackinder *et al*. [77]) and the discovery of novel genes possibly involved in calcification and coccolithogenesis by using EST approaches, suppression subtractive hybridization, long serial analysis of gene expression, microarrays for gene expression analysis and quantitative RT-PCR [11], [75], [76], [85], [86], [87], [88], [89], the details of the process of and the genes involved in coccolith formation in *E. huxleyi* are still unknown.

Genes potentially involved in calcification like carbonic anhydrase or the calcium-binding glycoprotein with a high glutamic acid, proline, and alanine content (GPA) [88], [90], [91] were not detected as calcifying factors in our study. This indicates that these two genes might be regulated at the transcript level or they fulfill cell-biological tasks in the non-calcifying life-cycle stage as well, as also indicated recently by Dassow et *al*. [75] and Rokitta et *al*. [76].

*Emiliania huxelyi* is known for its flexible responses in eco-physiological studies [49]. In particular, recent studies on carbonate chemistry changes showed strain-specific sensitivities to acidification of seawater [49], [50] which might be due to genetic variability described here. However, even with the same strain the diploid stage 1916 and the haploid 1917 exhibit different strategies and gene sets to acclimate to changing environmental conditions [89].

The genes possibly involved in virus susceptibility and calcification identified in this study provide targets for future studies on their expression, e.g. under virus attack, and for gene knock-out experiments.

## Methods

### Strains and culture conditions

*Emiliania huxleyi* strains (Table 1) and *Gephyrocapsa oceanica* were cultured in f/2 medium and *Isochrysis galbana* in K media at 15°C with a 16:8 light-dark cycle and 150 µE m^−2^ s^−1^. Strains EH2 and NZEH were treated with 1000 µg/mL Kanamycin because they were too sensitive against the antibiotic mixture. All other cultures were treated with a mixture of Ampicillin, Gentamycin, Streptomycin, Chloramphenicol and Ciprofloxacin (Table 3). Antibiotic treatment took place over 10–12 days. After 5–6 days cultures grown in 200 mL treated with antibiotics were transferred to 800 mL antibiotic treated f/2 media. Five to six days later cells were harvested on 1.2 µm RTTP ISOPORE filters Millipore. Cultures were checked against bacteria with acridine-orange staining. Only samples with no observed bacteria were used for analysis, although we cannot reduce a highly reduced bacterial background.

**Table 3:** Antibiotic treatment mixture.

Antibiotic	Concentration in culture [mg/mL]
Ampicillin	0.05
Gentamycin	0.003
Streptomycin	0.025
Chloramphenicol	0.001
Ciprofloxacin	0.010

### Genomic DNA labelling

All steps were performed in technical triplicates in order to avoid methodological errors in the hybridisation patterns interpretation. Genomic DNA was isolated from the samples using Qiagen DNeasy Plant Mini Kit (Qiagen, Hilden, D) and then subjected to amplification according to Agilent’s protocol for oligonucleotide array-based CGH for genomic DNA (version 5.0, June 2007). Restriction digestion was performed with 200 ng of genomic DNA for 8 h at 37°C. Digested DNA from each test strain and species was labelled with Cy5-dUTP whereas *E. huxleyi* strain CCMP1516 was labelled with Cy3 as reference. Labelled DNA sample yields and dye incorporation efficiencies were assessed photometrical (Nanodrop ND-1000, PecLab). Specific activity (pmol dyes perµg genomic DNA) were calculated as [pmol perµL dye/µg perµL genomic DNA] from the results of photometry.

### Microarray hybridizations

Labelled samples were then co-hybridized with DNA of the reference *E. huxleyi* strain CCMP1516 in triplicates to Agilent oligonucleotide-based 44k custom-made microarrays. One Array contained 37880 different transcripts derived from the *E. huxleyi* CCMP1516 genome project conducted by the U.S. department of Energy Joint Genome Institute (http://www.jgi.doe.gov/) using the best gene model for each locus. Microarrays were designed with Agilent’s eArray online application tool version 5.0 (accession number of the array design at ArrayExpress: A-MEXP-1696; http://www.ebi.ac.uk/arrayexpress).

A self-versus-self hybridization was performed in triplicates for determining probe specificity, array reproducibility, and microarray feature uniformity. Hybridizations were done for 24 h with 20 rpm using a hybridization chamber (Agilent technologies). After hybridization, the microarrays were washed according to the manufacturer’s instructions (Wash with Stabilization and Drying Solution, Agilent Technologies).

### Data acquisition and analysis

Microarrays were scanned using a G25655B Agilent microarray scanner with 100% photomultiplier tube (PMT) settings for both channels and 5 µm scan resolution. Signal intensities were detected and normalized by Feature Extraction software version 9.5 (Agilent Technologies) using the GE protocol and matrix. Spots which were not well above background in the self-self hybridization were removed before further analysis. Results were first analyzed using the MeV software package from TIGR [92]. An ANOVA test was performed for all groups with an alpha-level of 0.01 and a standard Bonferroni correction for multiple testing. The average intensity from the significant genes of the triplicates was used for further analysis. Probes which did not hybridize in the self-self hybridisation of the reference strain were excluded from the analysis. The software GACK was used to determine the cut-off on a strain-by-strain basis, accounting for variation in strain composition and hybridization quality [43]. GACK uses an estimated probability of presence (EPP) as an assignment scale. The hybridization against *I. galbana* was excluded from the following analyses due to problems in cut-off determination (visually misplaced Gaussian, data not shown). The criterion for the presence of genes in the non-reference strains hybridizations was EPP>95%. A consensus dendrogram (neighbour-joining) from the matrix of assumed presence of genes, including bootstrap analyses, was computed with the ape-package [93] in the data analysis software R [94].

### Identification of genes regarding virus susceptibility and calcification

The reference strain (CCMP1516) and the two out-groups *G. oceanica* and *I. galbana* were excluded in this analysis. Strains were grouped according to their virus susceptibility (Table 2) and possession of coccoliths (Table 1). A lower limit for the estimated probability of “gene present” >95% (EPP>95%) was used to elucidate whether a lack of certain genes, copy number changes or sequence divergence between reference and tester strain may explain the different biological properties of virus susceptibility or possession of coccoliths. To identify genes involved in virus susceptibility and calcification, we filtered for combinations of present genes in the susceptible (or calcifying, respectively) group of species versus absent genes in the resistant (or non-calcifying, respectively) group. We relaxed the criterion of perfect matches to allow for one false negative or one false positive, respectively. The resulting genes were manually analysed using BLASTP [95], [96] similarity searches version 2.2.24+ against the NCBI non-redundant protein database and the SwissProt database and were compared with matches of Pfam families [97]. All similarity search programs were applied with default parameters. Original data files for all arrays were uploaded in MIAME format to ArrayExpress with accession number E-MEXP-2388 (http://www.ebi.ac.uk/arrayexpress).

## Supporting Information

Figure S1 Multiple alignment of Bax Inhibitor 1-like protein (BI-1) using Clustal W in BioEdit. The protein sequence of *E. huxleyi* (ID 198434) was analysed using blastp [95], [96] similarity searches version 2.2.26 + against the SwissProt database in its standard configurations. The alignment was done with the four hits Q94A20 (*Arabidopsis thaliana*), Q49P94 (*Vaccinia virus* Lister), Q9DA39 (*Mus musculus*), and O7488 (*Schizosaccharomyces pombe* 972h) with ClustalW [98] in BioEdit. BLOSUM 62 was used as similarity Matrix.
(TIFF)

Table S1 Table of pair wise overlaps of all *E. huxleyi* strains and *G. oceanica* in terms of common present genes (EPP>95%). Excel-file including strain name and number of overlaps between each strain, including the reference strain.
(XLSX)

Table S2 Table of strain-specific genes for the reference strain *E. huxleyi* CCMP1516. Excel-file including array-, gene- and protein-ID, html-link to the genome website of the reference strain *E. huxleyi* CCMP1516, and the description and function of the identified genes.
(XLS)

Table S3 Identified genes of *E. huxleyi* in respect to virus susceptibility. Excel-file including array, gene and protein- ID, html-link to the genome website of the reference strain *E. huxleyi* CCMP1516, description and function of the identified genes of the 16 *E. huxleyi* strains.
(XLS)

Table S4 Identified genes of *E. huxleyi* related to the production of coccoliths. Excel-file including array, gene and protein- ID, html-link to the genome website of the reference strain *E. huxleyi* CCMP1516, description and function of the identified genes of the 16 *E. huxleyi* strains.
(XLS)
