# Supplementary figures and images for: Genome Variations Associated with Viral Susceptibility and Calcification in Emiliania huxleyi
Source: PLoS One. 2013 Nov 19;8(11):e80684. doi: 10.1371/journal.pone.0080684 (PMC3834299; doi:10.1371/journal.pone.0080684)

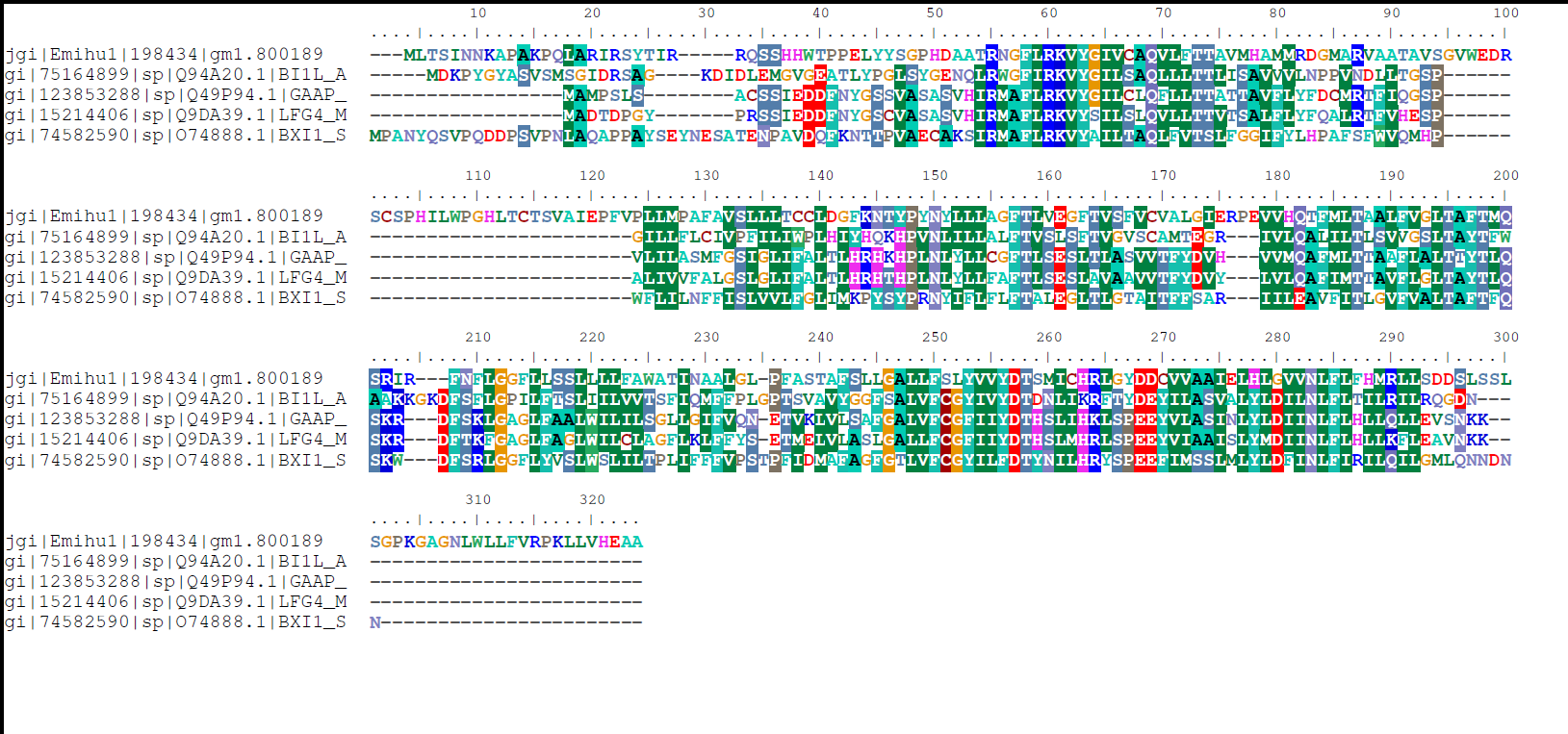

Supplement: Figure S1 — Multiple alignment of Bax Inhibitor 1-like protein (BI-1) using Clustal W in BioEdit. The protein sequence of E. huxleyi (ID 198434) was analysed using blastp [95], [96] similarity searches version 2.2.26 + against the SwissProt database in its standard configurations. The alignment was done with the four hits Q94A20 (Arabidopsis thaliana), Q49P94 (Vaccinia virus Lister), Q9DA39 (Mus musculus), and O7488 (Schizosaccharomyces pombe 972h) with ClustalW [98] in BioEdit. BLOSUM 62 was used as similarity Matrix. (TIFF) [file pone.0080684.s001.tiff]
